# A practical guide to acute pain management in children

**DOI:** 10.1007/s00540-020-02767-x

**Published:** 2020-03-31

**Authors:** Nan Gai, Basem Naser, Jacqueline Hanley, Arie Peliowski, Jason Hayes, Kazuyoshi Aoyama

**Affiliations:** 1 Department of Anesthesia and Pain Medicine, The Hospital for Sick Children, 555 University Ave, #2211, Toronto, ON M5G 1X8 Canada; 2 Program in Child Health Evaluative Sciences, SickKids Research Institute, Toronto, Canada

**Keywords:** Pediatric acute pain, Pain assessment, Acute pain service, Opioid, Multimodal management

## Abstract

In the pediatric population, pain is frequently under-recognized and inadequately treated. Improved education and training of health care providers can positively impact the management of pain in children. The purpose of this review is to provide a practical clinical approach to the management of acute pain in the pediatric inpatient population. This will include an overview of commonly used pain management modalities and their potential pitfalls. For institutions that have a pediatric acute pain service or are considering initiating one, it is our hope to provide a useful tool to aid clinicians in the safe and effective treatment of pain in children.

**Electronic supplementary material:**

The online version of this article (10.1007/s00540-020-02767-x) contains supplementary material, which is available to authorized users.

## Approach to pain

**Fig. 1:**
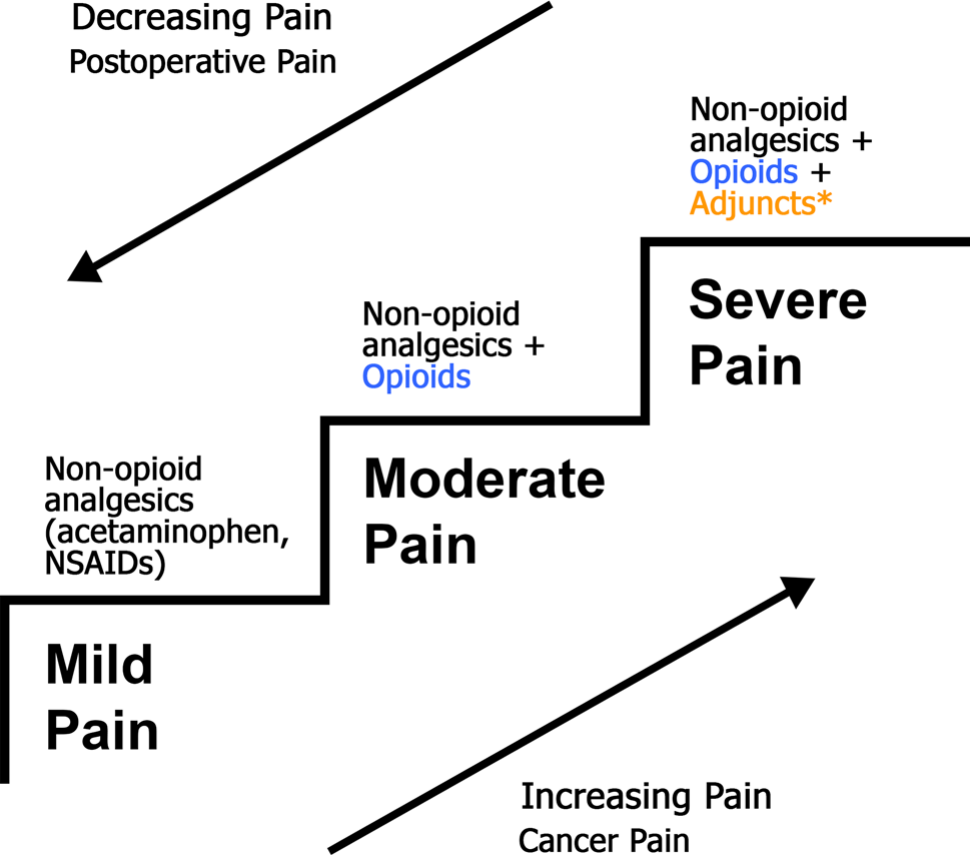
The World Health Organization (WHO) pain ladder modified for Acute Pain Management. ^a^Adjuncts include non-opioid analgesics such as ketamine, lidocaine, and gabapentinoids

Effective pain management is ideally practiced in a multidisciplinary model focusing on patient-centered care. The World Health Organization (WHO) [1] analgesic ladder provides a strong foundation for the treatment of pain that can be built upon to reflect more modern thinking and techniques around pain management (Fig. 1). Some of these modifications are presented in an updated WHO ladder with guiding principles on post-operative management of acute pain [2], which advocates 5 recommendations for the correct use of analgesics: (1) use the oral form of medication whenever possible, (2) analgesics should be given at regular intervals, (3) analgesics should be administered based on the severity of pain assessed using a pain intensity scale, (4) medication dosing should be tailored to the individual patient, and (5) attention to detail should be maintained throughout the prescription of pain medications.

## The acute pain service

The acute pain service (APS) is a specialized, multi-disciplinary inpatient team consulted to assist with management of severe pain. Within our institution, this team consists of a pediatric anesthesiologist, pediatric anesthesia fellow, clinical nurse specialist, and pediatric psychiatrist. The APS works in collaboration with the patient’s primary care team, bedside nurse, family, and pharmacists to provide a patient-centered multi-modal pain plan. Generally, the APS is consulted to assist in pain management when either a patient’s analgesic needs have grown beyond standard opioid dosing (Table 1) that their primary service is comfortable prescribing, or there is anticipated need for APS involvement for postoperative patients. Postoperative patients who automatically require APS management in our institution include those with an indwelling regional or neuraxial block catheter, patients who have received a single-shot peripheral nerve block, patients with a patient-controlled analgesia (PCA) technique, or patients receiving a Ketamine infusion.

**Table 1:** Opioid dosing summary for starting opioid doses, opioid equipotent conversion, and patient-controlled analgesia (PCA) dosing

Opioid	Morphine	Hydromorphone	Fentanyl	Oxycodone	Tramadol
Starting dose in opioid-naïve patients					
Oral	0.2–0.5 mg/kg PO/PR q4h	40–80 mcg/kg q3–4 h For children > 50 kg give 2–4 mg q3–4 h	N/A	0.1–0.2 mg/kg PO q4–6 h	1–3 mg/kg PO q4–6 h^a^ Maximum dose 400 mg daily
IV Bolus	50–100 mcg/kg q2h (max 3 mg)	10–20 mcg/kg q2–4 h For > 50 kg: 0.5 mg/dose (max 1 mg)	0.5 mcg/kg (max 50 mcg)	N/A	N/A
IV Infusion	10–40 mcg/kg/h	2–6 mcg/kg/h	0.5–2 mcg/kg/h	N/A	N/A
Equipotent dosing of opioids					
Equipotent PO dose	30 mg	6–7.5 mg	N/A	15 mg	180 mg
Equipotent IV dose	10 mg	1.5–2 mg	100 mcg	N/A	Unclear^b^
Oral to parenteral ratio	3:1	5:1	N/A	NA	N/A
Initial PCA dosing					
Concentration	1 mg/ml	200 mcg/ml	20 mcg/ml	N/A	N/A
Bolus dose (mcg/kg)	10–30 Start at 20	3–5 Start at 3	0.2–0.5 Start at 0.3	N/A	N/A
Basal infusion (mcg/kg/h) optional	4–30 Start at 10	3–5 Start at 3	0.15–1 Start at 0.2	N/A	N/A
2-h dose limit	80% of 2-h maximum	80% of 2-h maximum	80% of 2-h maximum	N/A	N/A

*PO* per Os (oral), *PR* per rectum, *N/A* not applicable

^a^Tramadol should be used with caution as it carries a US Food and Drug Administration (FDA) warning about use in children [24] because of reports of ultra-rapid metabolizers and severe respiratory depression

^b^The conversion between IV tramadol and other opioids is not well established in literature

## Assessment of pain

### Introduction

Management of pain remains undertreated in the pediatric population [3]. Additionally, it has been identified that pain may not be adequately or regularly assessed in pediatric patients admitted to hospital [3]. Appropriate, frequent, and clearly documented assessment of pain is vital to satisfactory pain management [4]. Self-report is preferred where possible because pain is a subjective experience [4]. When self-reporting may not be accurately relied upon in young or non-communicative children, additional assessment approaches such as behavior-based measures can aid in, or serve as an alterative to, self-reporting [4]. Reviewing physiologic parameters and reports from caregivers can round out the pain assessment.

### Pain assessment tools

Many methods of pain assessment exist, and ideally should combine patient and family history, assessments by the bedside nurse, physiologic parameters, and pain assessment tools. Assessment of pain includes both the measurement of pain severity, with a developmentally appropriate, validated tool, as well as a thorough pain history (exploring the pain quality, characteristics, location, onset, duration, aggravating and alleviating factors, and impact on function). The scales commonly used at our institution include the Numerical Rating Scale (NRS) [5], revised Face Legs Activity Cry and Consolability (r-FLACC) scale [6], revised Premature Infant Pain Profile (PIPP-R) [7], Faces Pain Scale—Revised (FPS-R) [8], and pain word scale (Table 2). Multiple tools are necessary to have appropriately targeted assessments for all age groups. The various cutoff values corresponding to mild, moderate, and severe pain are shown in Table 2.

**Table 2:** Summary of pain assessment tools

Tool	Target population	Scoring system	Scale
Numeric Rating Scale (NRS)	7 years and older	Ask the patient to assign a number to their pain, with 0 being no pain and 10 the worst pain ever	0–10 (mild 0–3, moderate 4–6, severe 7–10)
Faces Pain Scale—Revised (FPS-R)	5–12 years old	Picture-based scale where child selects 1 of 6 faces to represent their pain experience	0–10 (mild 0–3, moderate 4–6, severe 7–10)
Pain word scale	3–7 years old, or older children who are unable to use the NRS	Ask the child to quantify the severity of pain using words such as “none”, “a little”, “medium”, “a lot”	Descriptive words
Revised Face, Legs, Activity, Cry, Consolability (r-FLACC)	2 months–7 years old, or non-verbal/cognitively impaired patients of any age	5 behavior items each scored from 0 to 2 to a total of 10 points	0–10 (mild: 0–3, moderate: 4–6, severe: ≥ 7)
Premature Infant Pain Profile (PIPP-R)	Preterm and term infants	Combines 5 items (3 behavioral—brow bulge, eye squeeze, nasolabial furrow; 2 physiologic—heart rate, oxygen saturation) with gestational age	0–21 (mild: 0–6, moderate: 7–13, severe: 13–21)

Numerous pain assessment tools exist for the pediatric population. Currently, there is no evidence to recommend any one single tool as superior. One study has found that the r-FLACC [6] tool and Nursing Assessment of Pain Intensity (NAPI) have superior clinical utility compared to the Non-Communicating Children’s Pain Checklist-Postoperative Version (NCCPC-PV) in children with cognitive impairment [9]. For postoperative pain, the FLACC scale has been recommended for use in hospital [10]. Another study has shown that the r-FLACC is preferred by nurses because it is easy to use and pragmatic [11].

Pain assessment tools should not be the only method of quantifying pain. The pain score should be contextualized with assessment of patient satisfaction, family feedback, feedback from the patient’s nurse, and physiological parameters. This is especially true if pain scores flag a patient’s pain as moderate to severe. The patient should also be asked if the level of pain they are experiencing is tolerable to them, as some patients will report a pain score of 8/10 as being acceptable, while others will find it extremely difficult to cope with a pain score of 4/10. The pain assessment, therefore, should always be tailored to the individual patient and their own experience.

Ideally, patients at high risk for severe pain should have a pain assessment done by their nurse every 2–4 h. At minimum, every child admitted to hospital should have a pain assessment done every shift.

In our institution, patients followed by the APS team are seen, at minimum, once daily by the APS. For patients whose analgesia regimen has been modified during the morning visit, a second visit will take place in the afternoon to assess for effects of the modifications. The APS will also reassess patients on request by the bedside nurse or admitting team.

## Non-opioid adjunct medications

Consistent with ASA standards [12] and current practice, we advocate for multimodal analgesia approaches whenever possible. This includes optimization of non-opioid pain medications such as acetaminophen and non-steroidal anti-inflammatory drugs (NSAIDs) [13] if no contraindications exist. They should preferably be administered in a regular dosing schedule rather than as-needed. Some other commonly used non-opioid adjuncts employed within APS management include anti-spasmodics, Gabapentinoids, and intravenous Ketamine.

Patients who have undergone scoliosis repair often benefit from methocarbamol [14], a skeletal muscle relaxant, to help alleviate muscle spasms, which may not be alleviated by opioids. For teenagers, starting doses are typically 500–750 mg oral every 6–8 h as needed. If it is identified that muscle spasms are contributing to a significant part of the patient’s initial post-operative discomfort, methocarbamol can be administered regularly rather than on an as-needed basis for the first 2 days. The limiting side effect is drowsiness, and the dose should be decreased if drowsiness is significantly affecting the patient.

Lower limb orthopedic procedures may cause muscle spasms, especially for patients with cerebral palsy. For these patients, oral diazepam [11, 15] at a dose of 0.1 mg/kg (maximum dose 20 mg) orally every 6 h as-needed can be beneficial.

Ketamine is an *N*-methyl-d-aspartate (NMDA) antagonist with well-described analgesic properties [16]. It can be effective as an IV infusion either as the main analgesic agent or in combination with opioids and other analgesics. Typical analgesic doses are 2–4 mcg/kg/min as an intravenous infusion. The limiting side effect is dizziness or sedation. At these doses, hallucination or vivid dreams are usually not observed but should still be screened for during the APS visit.

In patients who complain of neuropathic pain (burning, tingling, numbness) or are at risk of neuropathic pain (i.e., based on possible nerve injury intra-operatively), the addition of an anti-convulsant in the form of a gabapentinoid can be helpful [17]. Two commonly used drugs are Gabapentin or Pregabalin. The main side effect limiting their use and dosing is drowsiness. Initial doses should be started low and titrated upwards. Starting doses of Gabapentin may be 5 mg/kg oral once a day at night-time, to be increased to twice daily the next day and then three times a day on the third day. In adolescents, typical starting doses are 300 mg orally, following the same schedule of increasing frequency on the first 3 days of use. Ideally, these patients should be discharged from hospital with a prescription to continue these drugs at home if they have been proven beneficial. Further dose adjustment or discontinuation should ideally be managed by a pain clinic. Pregabalin is typically tried only if patients have ongoing neuropathic pain that did not respond to gabapentin or could not tolerate gabapentin due to side effects.

## Non-pharmacologic treatment options

The multimodal approach to pain management also includes non-pharmacologic options. These include both physical and psychological strategies [4]. Patients may benefit from massage, heat compresses, ice packs, repositioning, or some physical activity (such as walking or sitting up in a chair for a short period of time). Some patients may find cognitive behavioral strategies, such as using imagery or relaxation, to be helpful. In pediatric patients, hypnosis has been shown to be effective for reducing pain [18].

When caring for patients with pain, it is also important to consider psychosocial factors that will impact how a patient experiences pain. It has been established that anxiety, catastrophizing, and depression can affect how a patient experiences pain and can aggravate or prolong acute pain [19–21]. Such factors as anxiety and mood should be screened for during APS visits, either by simple observation of the patient and their interactions, or from directed questions if there is suspicion for severe anxiety or depression. Subsequent strategies are tailored to each individual patient and severity of symptoms. Management may include giving the patient a chance to voice their concerns, validate their fears, and reassure them by reviewing their pain plan. For severe anxiety or depression, a psychiatrist may be consulted for pharmacologic or non-pharmacologic strategies.

## Systemic opioids

### Introduction

When a patient’s pain is established to be severe enough to require opioids, these drugs should be preferentially prescribed in oral or enteral form [4, 22, 23]. Anecdotally, the experience in our center has observed less side effects such as sedation, pruritus, and nausea after converting from intravenous to oral opioids. The severity of a patient’s pain should not prompt automatic use of intravenous opioids rather than an enteral form. Typically, immediate-release oral opioids for constant pain are ordered at 3–4-h intervals to minimize breakthrough pain and opioid side effects. Additional smaller doses can be available every 2 h for breakthrough doses. Patients with episodic pain should be ordered opioids only via an as-needed basis rather than regularly scheduled doses.

One of the major reasons to use an intravenous opioid rather than oral is concern regarding poor absorption or inability to administer drugs enterally. Examples would include patients who have inflammation or infections of the gastrointestinal tract (i.e., colitis, mucositis) or have poor bowel motility (i.e., postoperatively following bowel surgery, functional ileus, severe vomiting).

For patients with severe pain for whom an intravenous form of opioid has been planned, the opioid schedule should be matched to the type and duration of pain. Simplistically, patients with constant pain should be on an opioid infusion. The effect of a single bolus of intravenous opioid is relatively brief and should not be the sole regimen for patients with constant pain. Boluses should ideally be used on an as-needed basis for patients with sudden onset severe pain, or reserved for breakthrough pain for patients already receiving an opioid infusion.

We recommend the starting opioid in opioid-naïve patients should be morphine or hydromorphone. In our experience, a significant clinical difference between these two opioids has not been appreciated. A newer drug, Tramadol, has been used in adults as well as children, but should be prescribed with great caution. The US Food and Drug Administration (FDA) issued an updated warning about codeine and tramadol use in children in 2017 [24] because of reports of ultra-rapid metabolizers and severe respiratory depression. We would not recommend fentanyl as an initial opioid of choice given its increased potency and shorter half-life. Anecdotally we have observed tachyphylaxis in patients receiving fentanyl infusions.

### Approach to common opioid side effects

Some frequently occurring opioid side effects are summarized in Table 3. A common approach that can be applied to almost all opioid-related side effects is to maximize opioid sparing techniques (non-opioid analgesics, regional blocks, non-pharmacologic modalities) and consider opioid rotation. For conversion between different opioids, many opioid conversion tables have been published. We would refer the reader to Table 1, or the Canadian Guideline for opioid therapy and chronic noncancer pain [25, 26].

**Table 3:** Common opioid-induced side effects

Side effect	Interventions
Pruritus	Ensure PRN^a^ anti-pruritics are always available for the patient taking opioids (Diphenhydramine) Additional anti-pruritics: cetirizine Decrease opioid dose if pain is very well managed Rotate opioid Naloxone infusion (0.25–1 mcg/kg/h)
Nausea, vomiting	Ensure PRN anti-emetics are always available for the patient taking opioids (options: Ondansetron, Dimenhydrinate, Metoclopramide, Prochlorperazine) Decrease opioid dose if pain is very well managed Rotate opioid
Respiratory depression	Apnea, severe hypopnea or severe desaturation events—stop opioid, supplemental oxygen or bag mask ventilation, consider naloxone, call for immediate attention of anesthesiologist or critical care services For moderate respiratory depression without immediate oxygenation or ventilation compromise, titrate down opioid dose and continue to closely monitor patient for improvement of respiratory status
Sedation	Decrease opioid dose if pain is very well managed Rotate opioid If patient tolerates enteral route, switch from intravenous to enteral route
Constipation	Decrease opioid dose if pain is very well managed Promote physical activity (standing, walking) as tolerated by patient Stool softener, laxatives
Opioid-induced tolerance	Consider opioid rotation Add non-opioid analgesic (i.e., ketamine)

*PRN* pro re nata (as needed)

### Opioid withdrawal

Opioid withdrawal has been reported in patients who have been receiving regular opioids for as brief as 5 days, but typically most patients who have been receiving opioids for less than 7 days do not suffer from withdrawal [27]. For patients who have been administered opioids for 7 days or greater, a 10–20% of initial dose down-titration (or “wean”, per day) should be followed, modified by daily assessments for adequate pain control and presence of withdrawal symptoms. Common opioid withdrawal symptoms include (but are not limited to): irritability, anxiety, agitation, fever, sweating, and tachycardia. Patients who start exhibiting signs suggestive of opioid withdrawal should have their opioid wean held and possibly rewind their wean to the last dose where no withdrawal signs or symptoms were apparent.

Assessment for opioid withdrawal should be aided with a validated scoring tool. Our institution uses the Withdrawal Assessment Tool Version 1 (WAT 1) [28] to screen for opioid withdrawal and to provide a trend during any opioid wean (Online Resource 1).

## Patient-controlled analgesia (PCA)

### Introduction

Patient-controlled analgesia (PCA) with intravenous opioids is a commonly used modality for acute pain management in the pediatric population [29]. It allows for patients to self-administer small opioid doses. The delivery of this modality involves a computerized infusion pump pre-programmed to administer a set bolus dose of opioid when the patient presses a button. If appropriate, a background continuous infusion dose of opioid can also be programmed. Benefits of PCA use include improved pain scores and patient satisfaction compared to non-patient controlled forms of opioid administration [12, 30, 31].

PCA use is generally accepted as safe in the pediatric population [30, 32, 33]. It can be offered to any child who is able to grasp the concept of pressing a button to help relieve pain [34]. Typically, for institutions with an age requirement for PCA use, PCA can be used for children 6 years and older [29]. Although opioid consumption has been found to be higher with PCA compared with non-patient controlled regimens, generally severe adverse effects have not been found to be higher with PCA [30, 32]. PCA use has been associated with higher rates of pruritus [30] compared to conventional opioid administration.

Successful use of PCA as a method of patient-centered pain management requires adequate education of patients, families, and nursing staff. The concept of pushing the button by the patient specifically for pain, reassurance regarding safety measures, and teaching around potential opioid-related side effects should be discussed prior to the initiation of a PCA.

### PCA dosing

The PCA pumps we typically use can be programmed with a bolus dose, lockout time, basal (background) infusion dose, and 2-h dose limit (Table 1). The 2-h dose limit when starting a PCA is generally calculated as 80% of the maximum allowable 2-h dose. The majority of PCAs are administered without a background infusion. There is currently no strong evidence to favor using any one opioid over another for PCA purposes [31].

The use of PCA with a background infusion is more common in pediatric compared to adult practice [29]. In adults, PCA use with a background infusion compared to PCA without a background infusion has been shown to lead to a higher consumption of opioid without any benefits of improved analgesia or decreased side effects [12]. One meta-analysis examining PCA with a background infusion in postoperative pediatric patients did not find significant differences in pain scores, opioid consumption, or adverse events compared with PCA without a background infusion [35]. In our institution, for patients with a background infusion included in their PCA, there is a daily reassessment to wean or discontinue the background infusion to minimize opioid-related side effects. Ideally for immediately postoperative patients, we aim to discontinue the background infusion by postoperative day one or two.

Some common indications for initiating a PCA pump with a background infusion include:

1. Scoliosis repair postoperative pain management
2. Severe chemotherapy-induced mucositis pain
3. Severe sickle cell crises
4. Other severe constant pain requiring a background infusion based on APS assessment

The dosing is continuously re-evaluated by the APS, not only to optimize pain management and side effects, but also with the goal of weaning the opioid or converting to an enteral form when the patient is able to tolerate an enteral route. The additional benefit of converting to an oral form of opioid is that it allows patients to be discharged home if they still require opioids.

### Troubleshooting PCA-specific issues

Typical issues that arise during PCA management involve inadequate pain management or side effects of opioids (Table 3). In addition to the usual measures of pain assessment, the ratio of PCA demands to delivered doses can be calculated. This may be calculated on a per-hour basis or across a longer period of time such as 8 h. Ideally, patients should have a ratio of 1. Ratios greater than 2 have been suggested to predict more poorly controlled pain or need for opioid rotation [36]. Ratios greater than 2 may reflect inadequate pain control, improper PCA use, or insufficient PCA settings. We would caution that in some cases, a ratio greater than 2 may not indicate inadequate pain control [37], but rather need for re-education and discussion on why the patient has been pressing the button excessively. Some common practical problems seen in PCA management are described in Table 4, along with suggestions for resolving them.

**Table 4:** Patient-controlled analgesia (PCA) troubleshooting

Problem	Causes	Intervention
Patient not pressing button despite pain	Fear of pressing button (often related to potential opioid overdose or addiction potential)	Discussion with patient and family regarding reason for not using PCA and reassurance and education where appropriate
Patient over-pressing button	Misunderstanding of appropriate indications for pressing button (i.e., some patients feel inclined to press the button despite having little pain, or press the button whenever they see the green light on the PCA button indicating the lockout interval is finished)	This may require re-education regarding appropriate use of the PCA (some children feel that they should be pressing the button as much as possible despite actually having very little pain) or inactivating the light indicator on the PCA button (device-specific)
	Pressing for indications other than pain (i.e., for euphoric effects of opioid, anxiety, or sleep)	May not be appropriate candidate for PCA. Consider alternative method
Inadequate pain control despite appropriate use	The bolus or background dose may be too low, or the lockout interval too long	Assess for whether the bolus doses are effective and whether the effect lasts the 6 min of lockout. If there are no concerning side effects, increase bolus dose by 10–20% or decrease lockout to 5 min. Ensure non-opioid adjuncts are available
	Specific opioid may not be effective for this patient	Consider opioid rotation
	Assess for non-nociceptive pain (neuropathic, muscle spasms)	Institute adjuncts for neuropathic pain (consider gabapentinoids) or muscle spasms (methocarbamol, diazepam) or other non-opioid adjuncts (ketamine)
Over-sedation	Relative over-dose of opioid	Decrease bolus or background dose by 10–20%

### PCA weaning

**Fig. 2:**
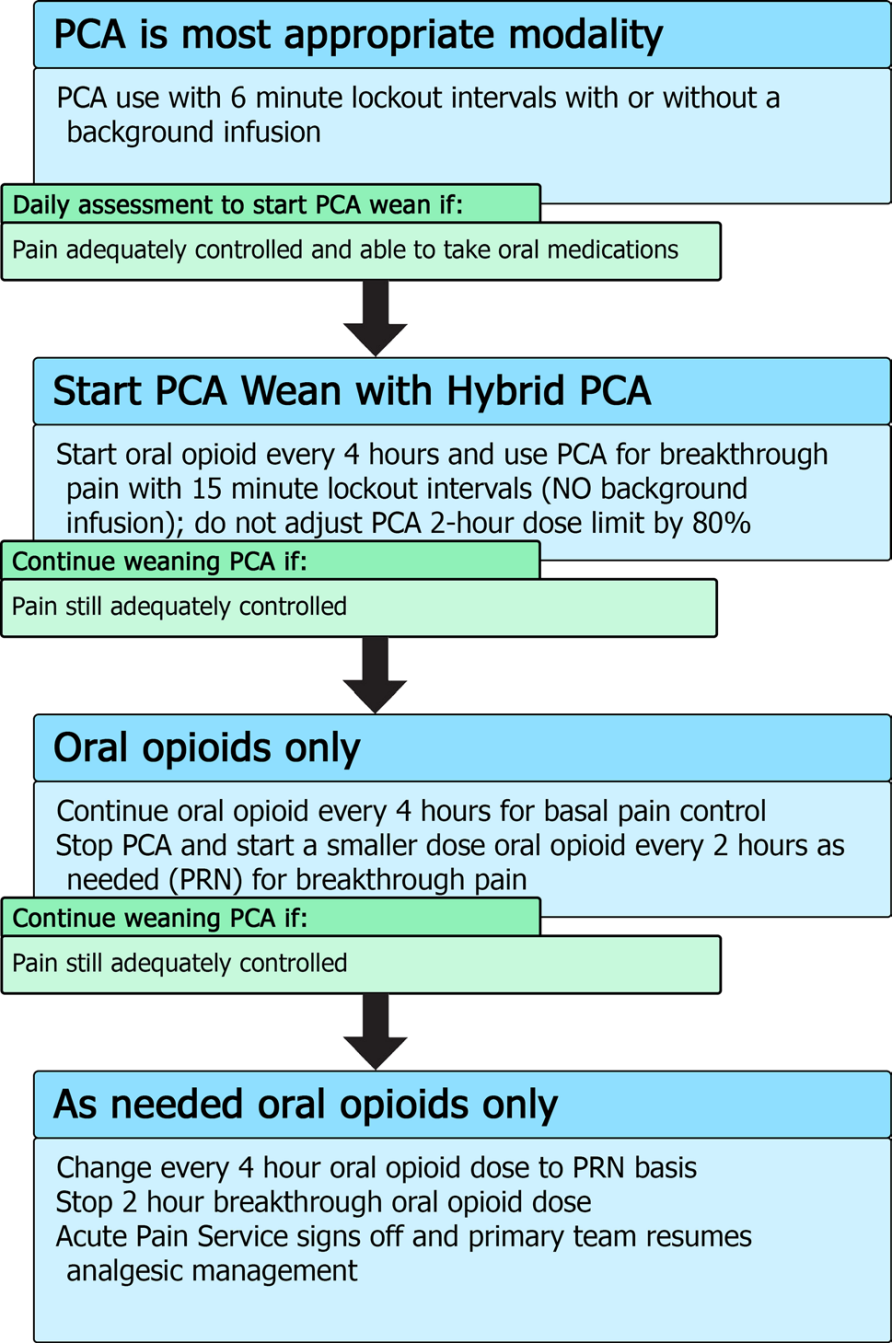
Summary of Patient Controlled Analgesia (PCA) wean with hybrid transition process. For patients using a PCA, there should be a daily assessment to determine if it is appropriate to consider weaning the PCA, especially if the patient is able to tolerate oral medications. The first step to weaning off a PCA includes starting a “hybrid” PCA set-up with the oral medication acting as a background opioid and the PCA being used for breakthrough. If this hybrid is adequately treating pain, further weaning can take place by stopping the PCA completely and using oral breakthrough doses instead

Patients should be converted from PCA administration to an enteral form of administration once the patient is able to tolerate enteral routes and has acceptable pain control (Fig. 2). Patients maintained on a PCA may also self-wean their opioid intake as their pain improves and they do not press their button as frequently. For patients who have been on long-term opioids (i.e., greater than 7–14 days of regular opioid use [27]), there is risk of opioid withdrawal syndromes manifesting if opioids are weaned too quickly. Occasionally, for patients who have greatly improved pain and essentially stop pushing their button, we have added a continuous background PCA infusion that can be weaned on a daily basis by 10% to avoid precipitating withdrawal.

For patients who continue to require opioids and are able to tolerate oral intake and, thus, can be treated with oral medications, we use a “Hybrid PCA” modality that combines oral medications with PCA for breakthrough pain in the process of weaning a patient off their PCA (Fig. 2). This allows patients the ability to continue to titrate their opioid usage to their pain while at the same time starting them on an oral form of opioid to allow more stable plasma concentrations of drug (see Table 1 for conversion doses).

For hybrid PCA dosing, the same bolus dose can be used but with an increase of the lockout interval to 15 min. There is no background PCA infusion as the basal opioid intake should be in the form of the oral medication. The 2-h maximum dose will be 100% of the 2-h calculated maximum dose (rather than adjusted to 80%). If, after a day of the hybrid PCA, the patient’s pain is still well controlled, the PCA is stopped completely and the breakthrough dosing is converted to an oral opioid dose on an as-needed basis. On the next day, if the pain is still well controlled with little use of breakthrough doses, the regularly dosed opioid can be converted to an as-needed basis only. Typically, most pediatric services are then able to manage such an oral opioid regimen and the APS signs off.

For a typical case example in the APS management of a PCA, please see Online Resource 2.

## Epidurals

### Introduction

Epidural analgesia is a safe and effective method of managing pain in children [38–40]. Currently, the literature surrounding epidural use in children is lacking when compared with adult studies. In adults, numerous trials have shown superior pain relief when comparing epidural techniques to oral or intravenous opioids, or other alternative approaches [12, 41–45]. In abdominal surgery, epidural local anesthetic hastens the return of bowel function and decreases postoperative pain [46].

The focus of this review is not to discuss epidural insertion techniques, but instead to discuss managing epidural catheters and infusions on the acute pain service.

### Epidural solutions

At our institution, the available solutions for continuous regional block infusions are all bupivacaine based with the options to add epinephrine and/or fentanyl. Table 5 summarizes the typical starting doses for epidural infusions within our institution. Alternative local anesthetics include ropivacaine and levobupivacaine, both of which are less cardiotoxic than racemic bupivacaine [47]. We currently do not offer patient-controlled epidural analgesia. Generally, most patients are started on epidural infusions using 0.1% bupivacaine with epinephrine, with more dilute concentrations used initially if there is concern from surgeons about being able to accurately assess neurologic function distally. The addition of an epidural opioid is not as standardized and is decided on a case-by-case basis. Some APS physicians choose not to add opioid into the epidural solution if there will be a second administration source of opioid, as this can confound the ability to titrate opioids with two concurrent sources. This second source may be in the form of intrathecal, oral, or systemic opioids. Two simultaneous sources of opioids may increase the risk of opioid side effects, making it more challenging to titrate to desired clinical effect.

**Table 5:** Epidural solutions typically used at The Hospital for Sick Children

Epidural type	Solution components			Dosing		
	Bupivacaine concentration (%)	Epinephrine concentration	Fentanyl concentration (mcg/ml)	Rate (ml/kg/h)	Bupivacaine (mg/kg/h)^a^	Fentanyl (mcg/kg/h)
Lumbar/caudal	0.125	1:400,000	0–2	0.16–0.32 (max 18 ml/h)	0.2–0.4	0.16–0.64
	0.1	1:500,000	0–2	0.2–0.4 (max 18 ml/h)	0.2–0.4	0.2–0.8
	0.0625	1:800,000	0–2	0.32–0.64 (max 18 ml/h)	0.2–0.4	0.32–1.28
Thoracic	0.125	1:400,000	0–2	0.1–0.16 (max 10 ml/h)	0.125–0.2	0.1–0.32
	0.1	1:500,000	0–2	0.1–0.16 (max 12 ml/h)	0.1–0.16	0.1–0.32
	0.0625	1:800,000	0–2	0.16–0.2 (max 14 ml/h)	0.1–0.125	0.16–0.4

^a^For neonates and infants, the bupivacaine infusion dose range is 0.2–0.3 mg/kg/h. Reduce infant infusion rates by at least 30–40% after 48 h

### Epidural assessments

All patients with an epidural infusion will have a daily assessment by the APS. The ideal epidural analgesia will provide satisfactory pain relief while minimizing side effects. Common issues seen with well-working epidural infusions include a dense motor block for lumbar epidurals, and can be distressing to older children. Daily epidural assessments should include not only an assessment for adequate pain control, but also screen for potential adverse effects, including assessments of the epidural insertion site and gross neurologic function. Some commonly observed issues encountered in epidural management are summarized in Table 6.

**Table 6:** Common problems encountered when managing epidural catheters

Problem	Neurologic testing	Causes	Interventions
Inadequate pain control	Appropriate dermatomes numb on ice-test	Epidural solution too dilute	Use more concentrated local anesthetic solution Add epidural opioid if none used Supplement with systemic opioids
	Unilateral block	Inadequate epidural solution spread Catheter migration towards one side	Bolus with epidural solution to increase spread of block Withdraw catheter 1 cm Reposition patient (ensure non-blocked side dependent)
	Some dermatomes are numb but not all the targeted dermatomes	Epidural and incision mismatch Inadequate epidural infusion volume	Consider a bolus of epidural solution Increase the continuous infusion rate (by 10–20%) Add epidural opioid if none used
	No dermatomes blocked	Epidural failure (catheter is not in the epidural space)	Remove epidural catheter after ensuring no contraindications to immediate removal Start alternative analgesic regimen
Dense epidural blockade	Target dermatomes numb with or without motor block	Epidural solution too concentrated	Counsel patient and parents on expected numbness sensation of working epidural Use less concentrated local anesthetic solution, especially if significant motor block
	Target dermatomes plus additional dermatomes blocked	Epidural solution volume too high	Decrease epidural solution infusion rate
Severe back pain	Assess for dense motor block or neurologic deficit	Must rule out infection or hematoma	Assess epidural insertion site for signs of infection Consider emergent magnetic resonance imaging (MRI)
Fever	Assess for dense motor block or neurologic deficit	Must rule out infection related to epidural	Assess epidural insertion site for signs of infection Labwork to perform infectious work-up (complete blood count, cultures) Consider removal of epidural catheter if ongoing fever and no other source of fever identified

### Epidural transition and removal

**Fig. 3:**
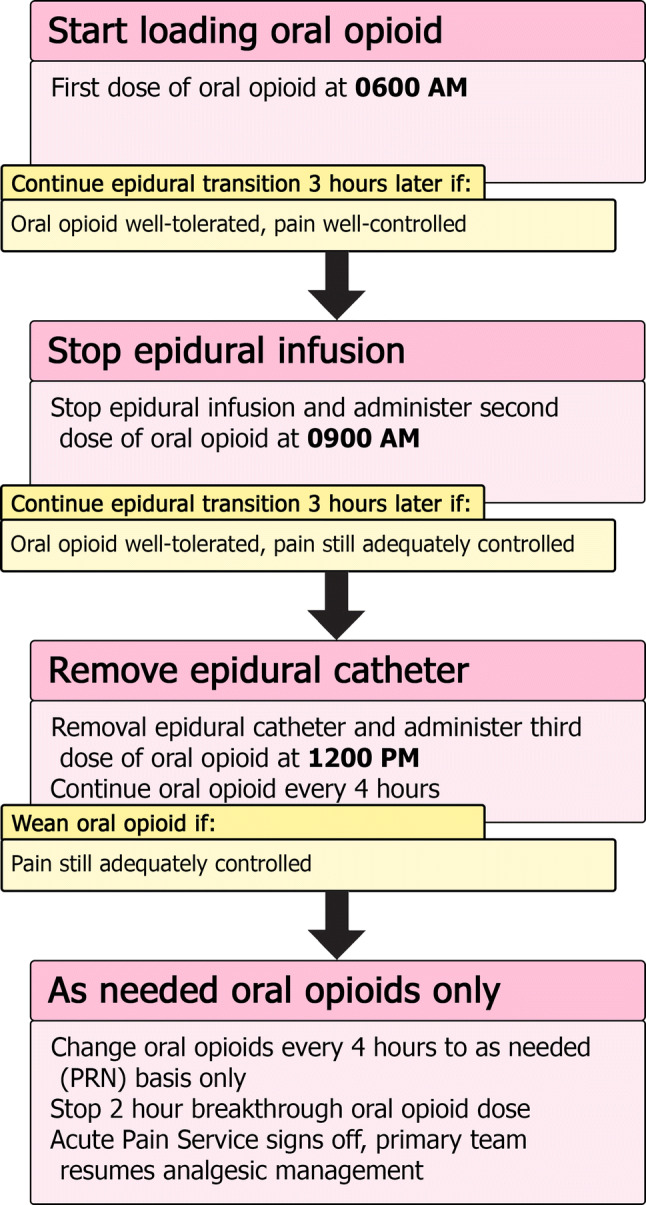
Summary of epidural transition to oral opioid process. Once it is determined the epidural can be discontinued and the patient can tolerate oral medications, the transition can be started. Typically, patients start their transition early in the morning to ensure that if troubleshooting needs to occur, it will be done during the daytime. The oral opioid is loaded with 3 doses given every 3 h. The epidural infusion is held once the second dose is given. If the patient’s pain is still well controlled, the epidural catheter is removed after the third dose. Continue regularly schedule oral opioid every 4 h until the next morning, when it can be assessed whether to convert the patient to oral opioid on an as-needed basis

Typically, our epidural catheters are removed after a maximum of 4–5 days. We also plan the epidural removal around any expected discharge planning timeline the surgical team has anticipated. One major concern for our patients and their families is to ensure that the transition from a well-working epidural to oral analgesia is as smooth as possible (Fig. 3). On the day of a planned epidural removal, the first dose of oral opioid is given early in the morning, while the epidural infusion is still running. The second dose occurs 3 h later at the same time the epidural infusion is held. If pain is still adequately managed 3 h later, the third dose of oral opioid is administered and the epidural catheter is removed at that point. Oral opioids are then continued every 4 h thereafter for a day, to be reassessed the next day. Should the pain not be managed adequately during this process, the dosing of oral opioids can be reassessed or additional breakthrough doses given. If the transition leads to severe pain, and the epidural can wait to be removed another day, consideration can be given to re-bolusing the epidural and trying the transition again the next day with a different regimen of oral opioids.

For a typical case example in the APS management of an epidural, please see Online Resource 3.

## Peripheral nerve blockade

Typically, peripheral nerve blockade is performed in our center as single-shot injections with occasional placement of peripheral nerve catheters. Any peripheral nerve block that has a motor block component will be followed by the APS until the day after block performance or nerve catheter removal. This is to ensure satisfactory recovery of nerve function as well as a smooth transition to oral pain medications. For procedures where severe pain is anticipated in the first day postoperatively with a single-shot block, regularly scheduled oral opioids can be initiated at 6–8 h post-peripheral nerve blockade to ensure loading of oral opioid prior to the nerve block wearing off.

For indwelling nerve catheters, much of the same principles in management of epidural catheters can be applied, with daily APS visits to ensure ongoing effective pain management, minimal side effects, and to assess the catheter insertion site for signs of infection or other concerning features. When planning the removal of a nerve block catheter with anticipation of ongoing significant pain, the same general approach of administering doses of oral opioid prior to stopping an epidural infusion can be applied to transitioning from a nerve block catheter (Fig. 3).

## Implementation of an acute pain service

For institutions considering the implementation of a new APS, we will describe the experience in our institution and issues to consider when planning and growing such a service. Initial planning should involve gathering data on current cases (i.e., major surgical operations) that might require APS management to assess whether there is the caseload to justify establishment of an APS. Furthermore, a survey of patients, families, and health care professionals involved in the postoperative care of patients undergoing procedures at high risk for postoperative pain should be undertaken to establish the current adequacy of pain management and look for need for an APS.

Once it is decided that there is a sufficient demand for an APS, a dedicated anesthesiologist should lead the team in establishing the APS within the hospital. It will be important to meet with hospital administrators to ensure their support of the establishment of an APS. A nurse with an interest in pain management should be involved, ideally with a background in care for post-operative patients, and be ready to advocate for the APS throughout the hospital and with nursing colleagues. More training should not be a mandatory requirement at the beginning, since much of the training can come from experience and working with the anesthesiology department. Clinically, there should be 24-h coverage of the APS by anesthesiologist services, but this does not necessarily need to be a dedicated APS anesthesiologist especially at nighttime and over weekends.

Nurse-based APS models are well established [48, 49], and may be a good cost-effective starting point, in which a dedicated pain nurse staffs APS during the daytime, with supervision from an anesthesiologist. The pain nurse plays a significant role in managing APS modalities and educating ward nurses and other staff around APS issues [50]. Overnight and weekend coverage can include a second nurse or anesthesiology trainee under supervision by a consultant anesthesiologist.

Foundational work should include multi-disciplinary discussion and writing of pain-related policies, such as minimum safety monitoring for various pain modalities (Online Resource 4). The equipment required will be that involved in placement of peripheral or neuraxial nerve blocks, and any infusion pumps to maintain continuous infusions. We would recommend the establishment of a registry or database for continuous quality improvement.

Once the groundwork has been established, and all the safety mechanisms and policies have been finalized, the clinical implementation of the APS should be launched in a stepwise approach, with a pilot program in a single patient population of similar surgical procedures. It would also be safer to initially start the program in older children. Ongoing quality improvement strategies such as a repeat survey to assess for improved pain control after APS implementation will help the service improve and expand to more patient population groups, and eventually throughout the hospital.

Although there will be costs associated with an APS service, there should not be great additional costs other than the aforementioned specialist time and some basic equipment for running of modalities such as PCA or nerve block infusions. One center estimated establishing APS in their institution at a cost of $3–4 USD per patient [49], however this did not include equipment costs. An effective APS could contribute to improved pain control, improved patient and family satisfaction, decrease opioid consumption, and decrease adverse effects associated with uncontrolled severe pain [51–54]. In our center, we also provide a significant educational role to nurses and medical services in multi-modal pain management. It has been argued that an effective APS can offer cost-savings [53, 55, 56], but given the multi-factorial effects and considerations involved with running an APS, this is hard to definitively determine.

In areas of low resource, pain management could be greatly improved with increased education, awareness, and training of practitioners in pain medicine [57, 58]. In such settings, a model that would likely be successful is improvement in the recognition and treatment of pain with standard modalities, such as morphine, rather than new techniques.

## Future research work and quality improvement

There is ongoing need for improvement in the management of acute pain in hospitalized pediatric patients. Future research and quality improvement could benefit from focusing on ensuring adequate training and standardization of pain assessment for all health care providers. Hospital protocols should include an assessment of pain with every nursing shift for each patient with clear documentation to allow for assessment of trends. Ongoing training in pain management should be implemented for all physicians who treat children. The treatment of pain in children should not be the sole focus of physicians on the pain service but of any health care providers who care for children. It would also be very valuable if a cost–benefit analysis of APS care could be measured, and this could give institutions who do not yet support an APS more reliable information with which to make future decisions.

## Conclusion

For a variety of reasons, pediatric pain is still under-treated in modern medicine. One identified problem has been limited experience and training in acute pain management for physicians treating children. It is our hope that by describing typical practices used in our institution, we can share our knowledge and experience with other professionals endeavoring to safely minimize painful experiences for children.

## Electronic supplementary material

Below is the link to the electronic supplementary material.

Supplementary file1 (PDF 871 kb)

Supplementary file2 (PDF 56 kb)

Supplementary file3 (PDF 54 kb)

Supplementary file4 (PDF 65 kb)
