# Supplementary material for: A practical guide to acute pain management in children
Source: J Anesth. 2020 Mar 31;34(3):421–33. doi: 10.1007/s00540-020-02767-x (PMC7256029; doi:10.1007/s00540-020-02767-x)
Supplement: Supplementary file 1 — Supplementary file1 (PDF 871 kb) [file 540_2020_2767_MOESM1_ESM.pdf]

A Practical Guide to Acute Pain Management in Children

Electronic Supplementary Material

Online Resource 1. Withdrawal Assessment Tool – 1 (WAT-1)

Journal of Anesthesia

Authors:

1. Nan Gai MD FRCPC
   Department of Anesthesia and Pain Medicine, The Hospital for Sick Children
2. Basem Naser MBBS FRCPC
   Department of Anesthesia and Pain Medicine, The Hospital for Sick Children
3. Jacqueline Hanley RN, BSc, MN
   Clinical Nurse Specialist, Department of Anesthesia and Pain Medicine, The Hospital for Sick Children
4. Arie Peliowski MD, FRCPC
   Department of Anesthesia and Pain Medicine, The Hospital for Sick Children
5. Jason Hayes MD, FRCPC
   Department of Anesthesia and Pain Medicine, The Hospital for Sick Children
6. Kazuyoshi Aoyama MD PhD
   Department of Anesthesia and Pain Medicine, The Hospital for Sick Children
   Program in Child Health Evaluative Sciences, SickKids Research Institute

Corresponding author:

Kazuyoshi Aoyama, MD, PhD

555 University Ave, #2211, Toronto, ON, Canada, M5G 1X8

1-416-813-7653

Online Resource 1. Withdrawal Assessment Tool - 1 (WAT-1).

WITHDRAWAL ASSESSMENT TOOL – 1 (WAT – 1)

© 2007 L.S. Franck and M.A.Q. Curley. All Rights reserved. Reproduced by permission of Authors.

Patient Identifier														
	Date:													
	Time:													
Information from patient record, previous 12 hours														
**Any loose /watery stools**	No = 0<br>Yes = 1													
**Any vomiting/wretching/gagging**	No = 0<br>Yes = 1													
**Temperature > 37.8°C**	No = 0<br>Yes = 1													
2 minute pre-stimulus observation														
**State**	SBS[1] ≤ 0 or asleep/awake/calm = 0<br>SBS[1] ≥ +1 or awake/distressed = 1													
**Tremor**	None/mild = 0<br>Moderate/severe = 1													
**Any sweating**	No = 0<br>Yes = 1													
**Uncoordinated/repetitive movement**	None/mild = 0<br>Moderate/severe = 1													
**Yawning or sneezing**	None or 1 = 0<br>≥2 = 1													
1 minute stimulus observation														
**Startle to touch**	None/mild = 0<br>Moderate/severe = 1													
**Muscle tone**	Normal = 0<br>Increased = 1													
Post-stimulus recovery														
**Time to gain calm state** (SBS[1] ≤ 0)	< 2min = 0<br>2 - 5min = 1<br>> 5 min = 2													
**Total Score (0-12)**														

WITHDRAWAL ASSESSMENT TOOL (WAT – 1) INSTRUCTIONS

- Start WAT-1 scoring from the **first day of weaning** in patients who have received opioids +/or benzodiazepines by infusion or regular dosing for prolonged periods (e.g., > 5 days). Continue twice daily scoring until 72 hours after the last dose.
- The Withdrawal Assessment Tool (WAT-1) should be completed along with the SBS[1] at least once per 12 hour shift (e.g., at 08:00 and 20:00 ± 2 hours). The progressive stimulus used in the SBS[1] assessment provides a standard stimulus for observing signs of withdrawal.

Obtain information from patient record (this can be done before or after the stimulus):

- ✓ **Loose/watery stools**: Score 1 if any loose or watery stools were documented in the past 12 hours; score 0 if none were noted.
- ✓ **Vomiting/wretching/gagging**: Score 1 if any vomiting or spontaneous wretching or gagging were documented in the past 12 hours; score 0 if none were noted
- ✓ **Temperature > 37.8°C**: Score 1 if the modal (most frequently occurring) temperature documented was greater than 37.8 °C in the past 12 hours; score 0 if this was not the case.

2 minute pre-stimulus observation:

- ✓ **State**: Score 1 if awake and distress (SBS[1]: ≥ +1) observed during the 2 minutes prior to the stimulus; score 0 if asleep or awake and calm/cooperative (SBS[1] ≤ 0).
- ✓ **Tremor**: Score 1 if moderate to severe tremor observed during the 2 minutes prior to the stimulus; score 0 if no tremor (or only minor, intermittent tremor).
- ✓ **Sweating**: Score 1 if any sweating during the 2 minutes prior to the stimulus; score 0 if no sweating noted.
- ✓ **Uncoordinated/repetitive movements**: Score 1 if moderate to severe uncoordinated or repetitive movements such as head turning, leg or arm flailing or torso arching observed during the 2 minutes prior to the stimulus; score 0 if no (or only mild) uncoordinated or repetitive movements.
- ✓ **Yawning or sneezing** > 1: Score 1 if more than 1 yawn or sneeze observed during the 2 minutes prior to the stimulus; score 0 if 0 to 1 yawn or sneeze.

1 minute stimulus observation:

- ✓ **Startle to touch**: Score 1 if moderate to severe startle occurs when touched during the stimulus; score 0 if none (or mild).
- ✓ **Muscle tone**: Score 1 if tone increased during the stimulus; score 0 if normal.

Post-stimulus recovery:

- ✓ **Time to gain calm state** (SBS[1] ≤ 0): Score 2 if it takes greater than 5 minutes following stimulus; score 1 if achieved within 2 to 5 minutes; score 0 if achieved in less than 2 minutes.

Sum the 11 numbers in the column for the total WAT-1 score (0-12).

[1]Curley et al. State behavioral scale: A sedation assessment instrument for infants and young children supported on mechanical ventilation. Pediatr Crit Care Med 2006;7(2):107-114.

SBS, State Behavioral Scale.

Reprinted from Pediatric Critical Care Medicine with permission. Original paper: Franck LS, Harris SK, Soetenga DJ, Amling JK, Curley MAQ. The Withdrawal Assessment Tool-1 (WAT-1): an assessment instrument for monitoring opioid and benzodiazepine withdrawal symptoms in pediatric patients. Pediatr Crit Care Med. 2008;9:573–80.
