# Supplementary material for: A practical guide to acute pain management in children
Source: J Anesth. 2020 Mar 31;34(3):421–33. doi: 10.1007/s00540-020-02767-x (PMC7256029; doi:10.1007/s00540-020-02767-x)
Supplement: Supplementary file 2 — Supplementary file2 (PDF 56 kb) [file 540_2020_2767_MOESM2_ESM.pdf]

A Practical Guide to Acute Pain Management in Children

Electronic Supplementary Material

Online Resource 2. Typical Case Example – Patient Controlled Analgesia (PCA)

Journal of Anesthesia

Authors:

1. Nan Gai MD FRCPC
   Department of Anesthesia and Pain Medicine, The Hospital for Sick Children
2. Basem Naser MBBS FRCPC
   Department of Anesthesia and Pain Medicine, The Hospital for Sick Children
3. Jacqueline Hanley RN, BSc, MN
   Clinical Nurse Specialist, Department of Anesthesia and Pain Medicine, The Hospital for Sick Children
4. Arie Peliowski MD, FRCPC
   Department of Anesthesia and Pain Medicine, The Hospital for Sick Children
5. Jason Hayes MD, FRCPC
   Department of Anesthesia and Pain Medicine, The Hospital for Sick Children
6. Kazuyoshi Aoyama MD PhD
   Department of Anesthesia and Pain Medicine, The Hospital for Sick Children
   Program in Child Health Evaluative Sciences, SickKids Research Institute

Corresponding author:

Kazuyoshi Aoyama, MD, PhD

555 University Ave, #2211, Toronto, ON, Canada, M5G 1X8

1-416-813-7653

Online Resource 2. Typical Case Example – Patient Controlled Analgesia (PCA)

The APS is consulted to see an 8 year old girl for help with pain control. She has a history of acute lymphoblastic leukemia currently receiving chemotherapy. She is experiencing oral pain as a result of chemotherapy-induced mucositis. Currently she is receiving analgesia in the form of an intravenous morphine infusion at 30 mcg/kg/h but has ongoing severe pain.

When she is seen by APS she reports constant background pain at 3/10 but occasional bursts of pain of 9/10. The pain has been getting worse since yesterday. There do not appear to be any opioid-related side effects such as nausea, pruritus, sedation, or respiratory depression. The option of a PCA to aid in the bursts of more severe pain is discussed with her and her family. She seems to understand the concept of pressing the button. She is started on a PCA with morphine with settings of 20 mcg/kg per bolus with a lockout of 6 minutes. Her background infusion is kept the same.

When she is seen the next day, her pain appears slightly better controlled but still inadequate. Over a 12-hour period, she pushed her PCA button 82 times but only received 40 deliveries of bolus doses (PCA demand to delivered ratio of 2.05). She is asked about the reasons for which she is pressing her PCA button, and her response is that it is for severe pain, which indicates appropriate use. Her constant pain remained at 3 out of 10 but her severe episodic pain has improved to 7/10. She still finds this pain too severe. Her PCA bolus dose is increased to 30 mcg/kg with the lockout interval and background infusion remaining the same. Later in the afternoon, when APS sees her again, her pain has not improved, but she feels more nauseated. Given the appearance of these side effects without any improvement in pain control, the option of opioid rotation to hydromorphone is discussed. Her parents report that she has previously been tried on hydromorphone but became more severely nauseated. After further discussion of options, it is decided that Ketamine will be added as an analgesic. The patient's bolus

morphine dose is reduced to 20 mcg/kg (to decrease the nausea) and a ketamine infusion at 2 mcg/kg/min is started.

The next morning, when the patient is seen by APS, she reports improved pain control. Her background pain is now rated at 2-3 out of 10. However, her episodic pain is now a 5/10. Her PCA demand to delivered ratio is 1.2. Although the patient continues to report pain, she does feel this is a level she is able to tolerate and feels vastly improved from yesterday as she is no longer nauseated. No further changes are made to her analgesic regimen. She is continued on these doses without further modification as her symptoms do not change over the next several days. A week later she begins to improve clinically and so does her pain. The decision is made to start weaning her analgesia. Her ketamine is weaned first. Since it is at the starting dose of 2 mcg/kg/min, it is simply stopped. She has been self-weaning her opioids slowly already as she has been pushing her PCA button less. She is continued for 24 hours on the same morphine infusion, and then is started on a 10% morphine wean per day to avoid precipitating withdrawal.
