# Supplementary material for: A practical guide to acute pain management in children
Source: J Anesth. 2020 Mar 31;34(3):421–33. doi: 10.1007/s00540-020-02767-x (PMC7256029; doi:10.1007/s00540-020-02767-x)
Supplement: Supplementary file 3 — Supplementary file3 (PDF 54 kb) [file 540_2020_2767_MOESM3_ESM.pdf]

A Practical Guide to Acute Pain Management in Children

Electronic Supplementary Material

Online Resource 3. Typical Case Example – Patient Controlled Analgesia (PCA)

Journal of Anesthesia

Authors:

1. Nan Gai MD FRCPC
   Department of Anesthesia and Pain Medicine, The Hospital for Sick Children
2. Basem Naser MBBS FRCPC
   Department of Anesthesia and Pain Medicine, The Hospital for Sick Children
3. Jacqueline Hanley RN, BSc, MN
   Clinical Nurse Specialist, Department of Anesthesia and Pain Medicine, The Hospital for Sick Children
4. Arie Peliowski MD, FRCPC
   Department of Anesthesia and Pain Medicine, The Hospital for Sick Children
5. Jason Hayes MD, FRCPC
   Department of Anesthesia and Pain Medicine, The Hospital for Sick Children
6. Kazuyoshi Aoyama MD PhD
   Department of Anesthesia and Pain Medicine, The Hospital for Sick Children
   Program in Child Health Evaluative Sciences, SickKids Research Institute

Corresponding author:

Kazuyoshi Aoyama, MD, PhD

555 University Ave, #2211, Toronto, ON, Canada, M5G 1X8

1-416-813-7653

Online Resource 3. Typical Case Example – Epidural

A 10 year-old 30kg male patient with cerebral palsy has undergone bilateral varus derotation osteotomies. A lumbar epidural was placed under general anesthesia in the operating room and the APS has been consulted for his postoperative pain management. He is started on a 0.1% bupivacaine and 1:500,000 epinephrine solution at a rate of 0.2 mL/kg/h (6 mL/h). He also has adjunctive medication such as acetaminophen, ketorolac, and breakthrough doses of morphine ordered. He is seen daily by the APS to assess for adequate analgesia and ensure there are no concerns with the epidural catheter.

When seen by the APS on postoperative day 1, the APS team is told by the bedside nurse and the patient's mother that they were not sure the patient's pain was well-controlled overnight. Being non-verbal, the patient is unable to self-report his pain. Overnight they noticed occasional grimacing and crying out, which is very unusual for him. He did manage to fall asleep but occasionally woke up crying. He did receive a dose of morphine orally which seemed to help slightly. On examination, he does not respond to an ice test for sensation to his lower extremities. His nurse does say that when they reposition him, he does not seem to grimace.

This is a challenging case because there is some evidence that the epidural is effective but the patient still appears to have episodes of pain. The APS decides to optimize the epidural first by increasing the infusion rate on the epidural infusion to 0.3 mL/kg/h and to reassess in the afternoon to allow for several hours to determine if this change was beneficial.

On the afternoon visit, about 6 hours later, the APS sees the patient again. Again, his mother and nurse report that the patient generally seems comfortable except for occasional episodes of signs of pain. Given that the epidural seems to be generally effective, consideration is given to muscle spasms as the source of discomfort. The patient is started on Diazepam oral 0.1 mg/kg (3 mg) every 8 hours as needed.

The next morning, both the patient's mother and nurse report that the night was much better. The patient received 2 doses of diazepam overnight, which seemed to be effective and minimized further grimacing or crying out. It is decided to continue diazepam orally for spastic pain and make no further changes if the pain remains well-controlled. The patient has an uneventful course for the next several days and seems to have well-controlled pain during repositioning and the start of physiotherapy.

On postoperative day 5, the patient is transitioned to oral morphine according to the transition protocol and his epidural catheter is removed after ensuring that his pain is still well-controlled. On postoperative day 6, the APS sees him for the last visit. The patient is still comfortable on oral morphine every 4 hours with occasional q2h breakthrough doses. There are no neurologic concerns with his lower extremities. The patient continues to have frequent sessions with the physiotherapist so the parents have requested that the regularly scheduled morphine be continued. No changes are made to the patient's opioid regimen and the APS team signs off. Further management and titration of opioids to an as-needed basis can be managed by the orthopedics team.
