# Supplementary material for: A practical guide to acute pain management in children
Source: J Anesth. 2020 Mar 31;34(3):421–33. doi: 10.1007/s00540-020-02767-x (PMC7256029; doi:10.1007/s00540-020-02767-x)
Supplement: Supplementary file 4 — Supplementary file4 (PDF 65 kb) [file 540_2020_2767_MOESM4_ESM.pdf]

A Practical Guide to Acute Pain Management in Children

Electronic Supplementary Material

Online Resource 4. Minimum monitoring and assessment recommendations for various pain modalities

Journal of Anesthesia

Authors:

1. Nan Gai MD FRCPC
   Department of Anesthesia and Pain Medicine, The Hospital for Sick Children
2. Basem Naser MBBS FRCPC
   Department of Anesthesia and Pain Medicine, The Hospital for Sick Children
3. Jacqueline Hanley RN, BSc, MN
   Clinical Nurse Specialist, Department of Anesthesia and Pain Medicine, The Hospital for Sick Children
4. Arie Peliowski MD, FRCPC
   Department of Anesthesia and Pain Medicine, The Hospital for Sick Children
5. Jason Hayes MD, FRCPC
   Department of Anesthesia and Pain Medicine, The Hospital for Sick Children
6. Kazuyoshi Aoyama MD PhD
   Department of Anesthesia and Pain Medicine, The Hospital for Sick Children
   Program in Child Health Evaluative Sciences, SickKids Research Institute

Corresponding author:

Kazuyoshi Aoyama, MD, PhD

555 University Ave, #2211, Toronto, ON, Canada, M5G 1X8

1-416-813-7653

Online Resource 4. Minimum monitoring and assessment recommendations for various pain modalities

Modality	Continuous Opioid Infusion	Patient Controlled Analgesia (PCA)	Epidural Infusion	Peripheral Nerve Block Infusion
**Available at bedside**	Self-inflating resuscitation bag, appropriately sized mask, oxygen, suction			
**Available on unit**	Naloxone	Naloxone	Naloxone<br>Diazepam<br>Atropine<br>Epinephrine	Naloxone<br>Diazepam<br>Atropine<br>Epinephrine
**Additionally required**			Vascular access<br>Foley catheter for lumbar epidural	Vascular access
**Continuously monitored**	Continuous oxygen saturation			
**Baseline assessment**	Vitals (HR, BP, RR)[a], sedation score[b], pain assessment			
**When starting or changing infusion, or on admission to unit if already infusing**	Vitals (HR, BP, RR), sedation score, pain assessment q1h[c] for 4 hours			
**Assessment during maintenance infusion**	RR, sedation score **q1h**<br>HR, BP **q4h**<br>Pain score **q4h** while awake		RR, sedation score **q1h**<br>Temperature, HR, BP, motor and sensory block level **q4h**<br>Pain score q4h while awake<br>Nerve block catheter and site assessment **q8h**<br>Ensure adequate patient padding to avoid sores, reposition patient **q2h**	
**Assessment following bolus**			Vitals (HR, RR, BP) **q5min** for 20 minutes	
**Assessment on discontinuing**			Motor and sensory assessment until patient returns to baseline function	
**General reasons to notify APS**	RR or Oxygen saturation fall below ordered parameters<br>Inadequate analgesia			
**Modality-specific reasons to notify Acute Pain Service**			Concerns regarding nerve block site, dressing, or catheter<br>Signs of local anesthetic toxicity<br>If patient is prescribed an anticoagulant	

[a]HR = Heart Rate, BP = Blood Pressure, RR = Respiratory Rate

[b]Sedation score at our institution: 0 = alert; 1 = occasionally drowsy, easy to arouse; 2 = frequently drowsy, easy to arouse; 3 = somnolent, difficult to arouse; S = normal sleep, easy to arouse

[c]q1h = every hour, q4h = every 4 hours, q8h = every 8 hours, q5min = every 5 minutes
